# Noble Metal Functionalized Metal Oxide Semiconductors for Enhanced Gas Sensing

**DOI:** 10.3390/molecules30244683

**Published:** 2025-12-06

**Authors:** Renqing Yao, Yi Xia, Li Yang, Jincheng Xiang, Qiuni Zhao, Shenghui Guo

**Affiliations:** 1 School of Metallurgical and Energy Engineering, Kunming University of Science and Technology, Kunming 650093, China; 20232202031@stu.kust.edu.cn (R.Y.); 20040051@kust.edu.cn (S.G.); 2 International Joint Research Center for Advanced Manufacturing Technology of Super Hard Materials, Kunming University of Science and Technology, Kunming 650093, China; 3 Research Center for Analysis and Measurement, Kunming University of Science and Technology, Kunming 650093, China; 18007287818@163.com

**Keywords:** noble metal, semiconducting metal oxide, chemiresistive gas sensor, electronic sensitization, chemical sensitization

## Abstract

Gas sensors are vital tools in areas such as environmental monitoring, industrial safety, and personal healthcare. Among various sensing materials, semiconductor metal oxides (SMOs) are widely studied owing to their high sensitivity, good stability, and notable catalytic activity. To overcome inherent drawbacks of pure SMOs—such as high operating temperatures, limited selectivity, sluggish response/recovery behavior, and inadequate long-term stability—functionalization with noble metals has emerged as a powerful modification strategy. This review systematically outlines the primary mechanisms through which noble metals enhance gas sensing performance and analyzes the key factors influencing sensor behavior. Finally, we discuss the current challenges and future directions in the development of noble metal-modified SMO gas sensors.

## 1. Introduction

Gas sensors play an important role in numerous fields. They aid in environmental protection by regulating gases such as carbon monoxide and ozone [1,2,3]. They detect hydrogen and methane leaks [4,5] and provide early warnings on hazardous gases such as hydrogen sulfide and ammonia in factories [6,7]. They assist in the breath examination in relation to illness in healthcare [8]. They are sensitive to formaldehyde and smoke in smart homes. Sensitizing these tools, enhancing them to be more accurate, fast, and reliable, is one of the ways to improve these issues, and new concepts of gas sensors are formed.

Gas sensors are available in various types such as electrochemical [9], optical [10], surface acoustic wave [11] and solid electrolyte-type [12] gas sensors. Resistive sensors with semiconductor metal oxides (SMOs) are one such type, and have been studied intensively because of their relatively easy preparation, low cost, large response signals, compatibility with integration and capability of miniaturization.

But unmodified single-metal oxide sensors do have significant limitations in real-world practice that limits their potential performance: lack of selectivity means that it is very hard to discriminate between specific target gases in mixed-gas streams, or in a humid environment; being operated at high temperatures not only increases power consumption, but also reduces their ability to maintain long-term stability; and the slow response/recovery times and susceptibility to environmental interference (especially to humidity) prevent their broader applicability.

As such, to overcome these drawbacks, researchers have examined a wide range of strategies, including optimization of the morphology and microstructure of metal oxides (e.g., the creation of nanomaterials and porous materials) [13,14,15], building heterojunctions between metal oxides [16,17,18], elemental doping [19], and functionalizing surfaces [20]. Notably, the deployment of noble metal nanoparticles—including Au, Pt, Pd, Ag, Rh, and Ru (Table 1)—has proven to be a highly promising route for the enhancement of SMO gas sensors, yielding multifarious and significant benefits to their sensing capabilities. The affinity of the noble metals enables adsorption, dissociation and surface reaction of target gas molecules which in turn makes the sensor more sensitive and have, for example, faster reaction/recovery dynamics. Moreover, electronic sensitization effects (e.g., formation of Schottky barriers at the noble metal–metal oxide interface) effectively control charge transfer and enhance resistance variations by gas interactions [5,21,22,23]. The chemical sensitization effect is especially significant, allowing specific noble metals (e.g., Pd with H_2_, Au with H_2_S) [24] to create selective bonds or a strong adsorbent with the particular target gases [25,26]; therefore, high sensor selectivity is significantly enhanced. Moreover, in the photo-assisted gas sensors, the surface plasmon resonance (SPR) effect of noble metals such as Au and Ag can significantly improve light absorption [27,28], which gives a significant argument to the promotion of the work of light-driven gas sensing.

As such, the functionalization of noble metals is crucial. It directly addresses the historic weaknesses of SMO sensors, their typically poor selectivity, requirement of high operating temperatures and slow response kinetics. In addition to resolving these fundamental problems, this strategy also paves the way to the creation of the new generation of high-performance sensors that are energy-efficient and highly specific.

**Figure 1:**
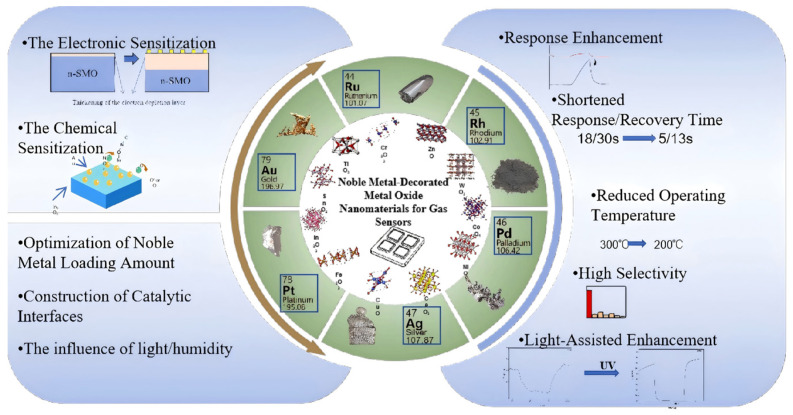
Schematic outline of this review.

A considerable number of existing review articles have provided insightful summaries of the research progress in noble metal-modified gas sensing materials from various perspectives [29,30], such as those focusing on specific noble metals or particular metal oxides. Building upon this foundation, this review aims to systematically integrate and synthesize the common mechanisms (e.g., electronic and chemical effects) of noble metal modification and their universal applicability across different material systems (e.g., oxides, sulfides). It will elaborately discuss the core mechanisms of noble metal modification and provide an in-depth analysis of key influencing factors, including the optimization of noble metal loading, the construction of catalytic interfaces, and the effects of conditions such as light illumination and humidity (Figure 1). The review also incorporates relevant work from our group on the Pt-MoS_2_ composite system [31], further validating the broad applicability of this modification strategy across different types of semiconductor materials. Furthermore, it explores performance optimization strategies in the context of various application scenarios and outlines future challenges and development directions in the field, hoping to offer a more systematic reference for researchers.

## 2. Intrinsic Properties of Noble Metals and Sensing Mechanisms

The oxygen adsorption model [32] is the most effective model that explains the operating mechanism of resistive-type solid-state metal oxide (SMO) gas sensors that mainly focus on reactions of the surface that occur between chemisorbed oxygen species with the target gas molecules [33]. In the case of n-type semiconducting oxides, including SnO_2_, ZnO and TiO_2_, the ambient air baseline electrical resistance is increased as a result of the capture of conduction electrons by adsorbed dioxin (O_2ads_) [34] forming surface ions (O^2−^, O^−^, etc.) [35,36] and creating an electron depletion layer at the sensor face. A surface reaction when the sensor is exposed to reducing gases (e.g., CO, H_2_) releases the previously trapped electrons; the reduction in the width of the depletion layer results in a measurable drop in resistance. On the other hand, encounters with oxidizing gases (e.g., NO_2_, O_3_) cause further withdrawal of the electrons, further depleting the depletion layer, thus adding to the sensor resistance [37,38,39].

In substances with p-type semiconductors like NiO and CuO, holes serve as the dominant charge carriers. The adsorption of oxygen in these materials tends to increase the concentration of holes creating a low-resistance baseline. Exposure to reducing gases causes the resistance to take the increase in form of holes consumed whilst that of oxidizing gases causes it to take the decrease in form of increased population of holes.

### 2.1. Electronic Sensitization Effect

**Figure 2:**
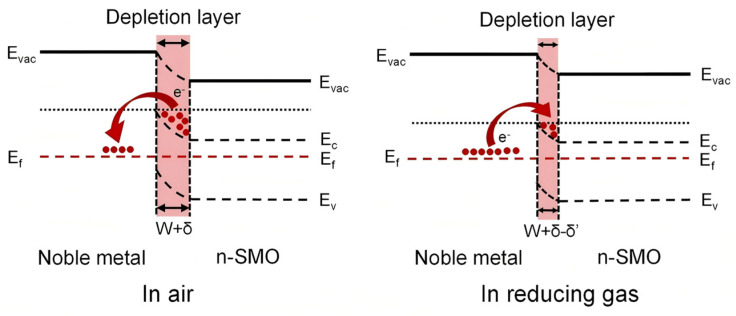
Schematic energy band diagram of noble metal-decorated n-SMO reproduced with permission from Ref. [30]. Copyright 2023, open access.

Noble metals, e.g., platinum (Pt), act as an important enhancement of the sensing properties of metal oxide semiconductor (MOS) sensors via the electronic sensitization effect [40,41]. This effect originates from the disparity in work function between the noble metal and the MOS; noble metals typically exhibit a higher work function (>5.0 eV) than n-type MOS materials (e.g., ZnO at approximately 4.2 eV), which leads to spontaneous electron transfer at the interface. Movement of electrons occurs as Fermi levels of the MOS-noble metal are matched until a Schottky barrier is created [42,43], and the electron depletion layer on the MOS surface is significantly increased [44] (Figure 2).

A fundamental aspect of electronic sensitization is that it does not scale the resistance under air (R_air_) and gas (R_gas_) environments equally. Instead, its primary action is to profoundly alter the initial electronic state by depleting the MOS of charge carriers, thereby establishing a very high R_air_. When a reducing gas is introduced, the ensuing surface reactions reduce the height of the pre-existing Schottky barrier. Crucially, this barrier modulation effect means that the relative resistance change (ΔR/R_air_) for a given quantity of released electrons is amplified. Although R_gas_ for the decorated sensor may also be higher than that of its pristine counterpart, the increase in R_air_ is disproportionately larger. This disproportionate increase is quantitatively reflected in the sensor response (for an n-type MOS to reducing gases, S = R_air_/R_gas_ − 1), leading to a greater R_air_/R_gas_ ratio and thus enhanced sensitivity.

For oxidizing gases, where the response is typically defined as S = R_gas_/R_air_ − 1, the impact of noble metal decoration is more complex and often dominated by catalytic sensitization. The noble metal catalyzes the dissociation of oxygen and the target gas, increasing the density of surface-adsorbed oxygen ions and leading to a dramatic rise in R_gas_. While the electronic sensitization effect also raises R_air_, the catalytic-driven increase in R_gas_ is usually dominant, resulting in a net enhancement of the response. However, in highly depleted nanostructures, the pre-existing low electron concentration due to electronic sensitization can sometimes limit the dynamic range for further electron trapping.

The extent of depletion layer broadening can be quantified using a physical model. The depletion layer width, *L_D_*, is related to the charge carrier concentration *n* and the built-in potential *V_bi_* by the following expression:

$$L_{D}=\sqrt{\frac{2\varepsilon_{0}\varepsilon_{r}V_{bi}}{en}}$$

where *ε_r_* denotes the relative dielectric constant of the material. Noble metal decoration increases *V_bi_*, thereby leading to a substantial expansion of *L_D_*. When *L_D_* approaches or exceeds the characteristic dimensions of the MOS structure (e.g., the thickness of a nanosheet), the charge carrier depletion can become nearly complete, resulting in a significant increase—by one to two orders of magnitude—in the baseline resistance of the sensor.

The electronic sensitization effect makes sensor responses much faster via band engineering of the noble metal–semiconductor interface, whose physical reality is the work function differentiated interfacial electron transfer and band modulation.

**Figure 3:**
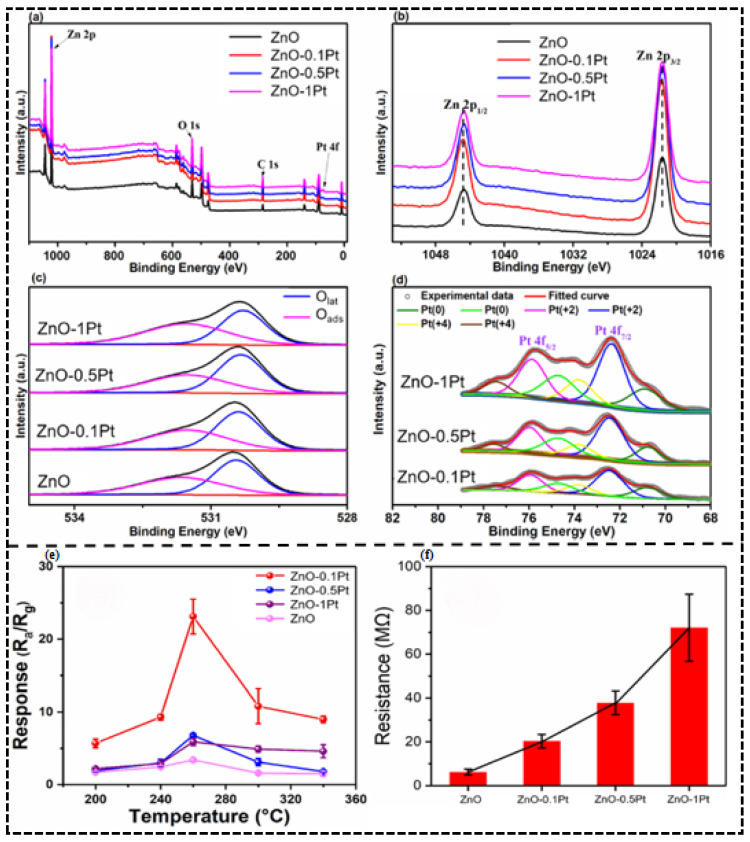
XPS spectra of pristine and Pt-loaded nanorods. (**a**) Survey spectrum and high resolution spectra of (**b**) Zn 2p, (**c**) O 1s, (**d**) Pt 4f. (**e**) Response of pristine and Pt-decorated sensors to 100 ppb of H_2_S at various temperatures. (**f**) Electrical resistances of pristine and Pt-loaded ZnO sensors in air at 260 °C. Reproduced with permission from Ref. [45]. © 2021 Elsevier B.V. All rights reserved.

The most common example of the electronic sensitization effect can be observed in the article by Zhou Li [45] et al. Pt-decorated ZnO nanorods (Figure 3a–d) were discovered as having a mixed assemblage of Pt^0^ and PtO (Pt^2+^/Pt^4+^ oxides) in this inquiry. The significantly lower working energy of an n ZnO compared to that of Pt(0) (5.65 eV) leads to thermodynamic equilibrium thus driving spontaneous electron transfer between ZnO and Pt^0^ thus creating a substantially thickened electron depletion layer on the ZnO surface, creating an abrupt increase in intrinsic resistance (Figure 3f). This interfacial electronic reconstruction has a great influence on the dynamic range of the gas response: the number of electrons released by the reaction of H_2_S with adsorbed oxygen (O^−^) effectively modulates the depletion barrier when the gas is exposed to a reducing agent, H_2_S, and the sensor is able to respond to 23.1 to 100 ppb H_2_S at 260 °C, a more than 580 percentage point improvement from pristine ZnO (response = 3.4) with a limit of 1.1 ppb.

**Figure 4:**
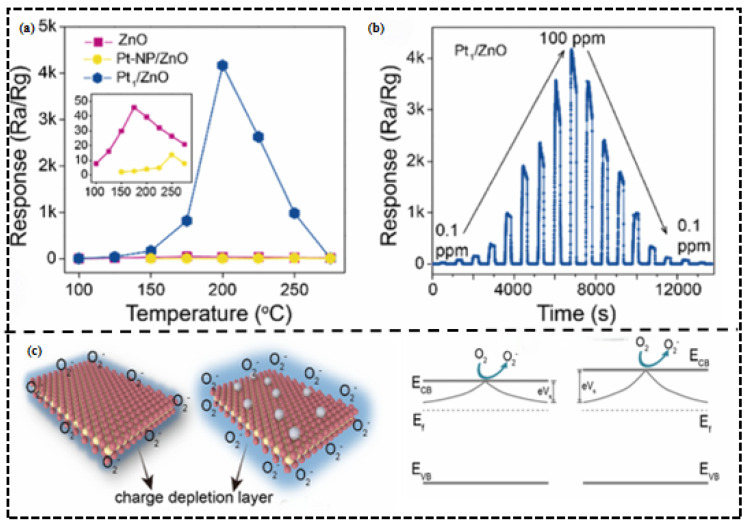
(**a**) Response of the three sensors to 100 ppm TEA at 100–275 °C (relative humidity: 40%). (**b**) Dynamic response–recovery curves of Pt_1_/ZnO sensor to TEA in concentrations of 0.1–100 ppm at 200 °C. (**c**) Band structure of ZnO and Pt_1_/ZnO. Reproduced with permission from Ref. [46]. Copyright © 2023, American Chemical Society.

In addition to the metallic modifications made during the basis of platinum, single-atom Pt-based modification is also evidenced of strong effects of electronic sensitization. In another study by Lingyue Liu et al. [46], single-atom Pt^2+^-modified ZnO performed remarkably. Pt, which consists of single atoms, facilitates electron transfer between ZnO and Pt^2+^ because of the work–function difference and makes the electron depletion line much more thick (Figure 4c). This increases the barrier height change caused by electrons released when triethylamine signal (TEA) reaction occurs leading to a 92-fold increase in response value (up to 4170) at 200 °C and a limit of 7.5 ppb (Figure 4b and Table 2).

### 2.2. Chemical Sensitization Effect

Chemical sensitization, also known as the “spillover effect”, centers on the catalytic role of noble metals (e.g., Pt [47], Pd [48], Au [49] (Table 3)) in optimizing the kinetics of chemical reactions on the metal oxide surface, rather than altering its bulk electrical properties. Unlike electronic sensitization, which directly modulates the depletion layer, chemical sensitization enhances performance via these pathways: Noble metal nanoparticles act as highly active sites that efficiently dissociate oxygen molecules to generate highly reactive atomic oxygen species (e.g., O^−^). These active species then “spill over” and diffuse onto the metal oxide surface, increasing the concentration of sites available for gas reactions. Concurrently, the noble metals significantly lower the activation energy barrier for the oxidation of target gases (e.g., ethanol, acetone). The synergy between these two effects drastically accelerates the entire kinetic cycle of “gas adsorption–surface reaction–electron transfer.” The ultimate manifestations are significantly faster response/recovery speeds, markedly improved sensitivity at low temperatures, and enhanced selectivity toward specific gases, as the reaction pathway is altered to promote deep oxidation.

**Figure 5:**
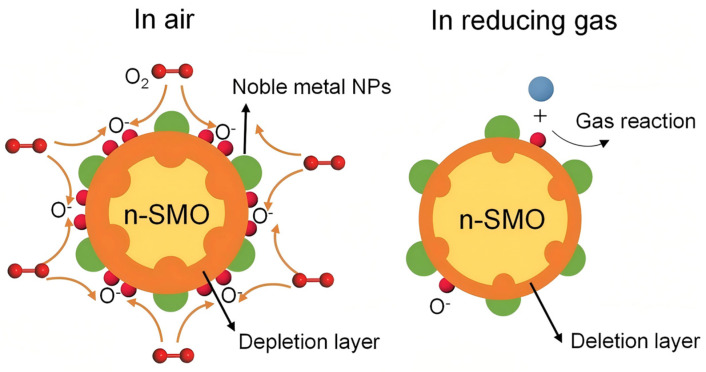
Schematic illustration of the chemical sensitization of noble metal-decorated n-SMO. Ref. [30]. Copyright 2023, Open access.

As illustrated in Figure 5, the process begins with the preferential adsorption and dissociation of O_2_ on the noble metal NPs. The resulting oxygen ions subsequently diffuse onto the SMO surface. When a reducing gas is introduced, this enlarged reservoir of O^−^ ions can react with a greater number of target gas molecules. This reaction releases electrons back into the conduction band of the SMO, leading to a rapid and substantial change in electrical resistance, which translates to enhanced sensor response.

**Figure 6:**
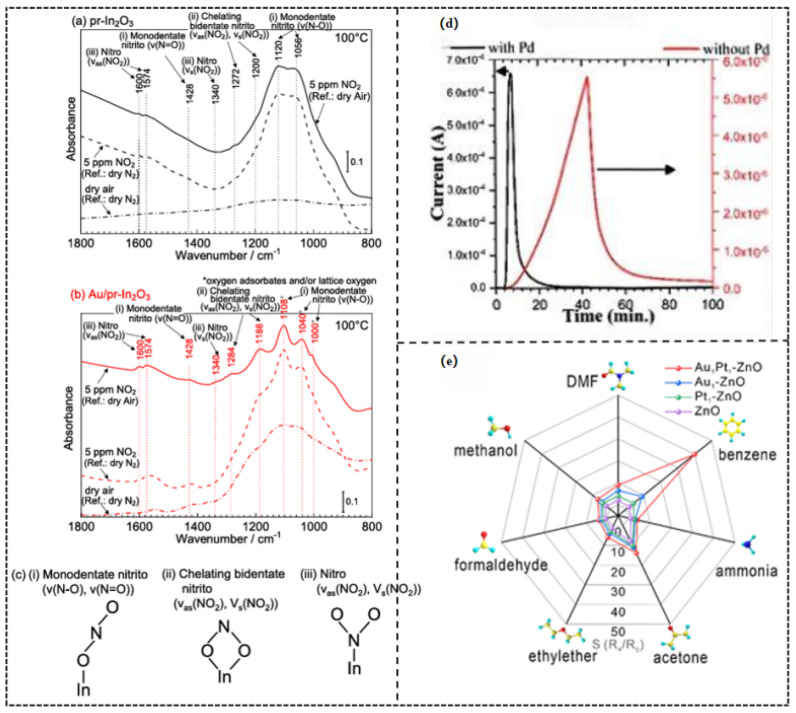
(**a**–**c**) DRIFT spectra of the (**a**) pr-In_2_O_3_ sensor and (**b**) Au/pr In_2_O_3_ sensor in 5 ppm NO_2_ in dry air at 100 °C (reference: in dry air and N_2_ at 100 °C) together with their spectra in dry air (reference: in dry N_2_ at 100 °C). (**c**) Illustration of (**i**) monodentate nitrito, (**ii**) chelating bidentate nitrito, and (**iii**) nitro. Reproduced with permission from Ref. [50] Copyright © 2021, American Chemical Society. (**d**) Dynamic sensor responses of Z_0.5_ with and without Pd catalysis at different operating temperatures. Reproduced with permission from Ref. [51] Copyright © 2022 The Authors. Published by American Chemical Society. (**e**) Responses of pure ZnO (350 °C), Au_1_-ZnO (350 °C), Pt_1_-ZnO (350 °C), and Au_1_Pt_1_-ZnO (300 °C) porous nanobelts to seven typical VOCs (50 ppm). Reproduced with permission from Ref. [52] © 2024 Elsevier B.V. All rights are reserved, including those for text and data mining, AI training, and similar technologies.

In a study by Taro Ueda et al. [50], in situ diffuse reflectance infrared Fourier transform spectroscopy (DRIFTS) analysis (Figure 6a–c) of porous In_2_O_3_ loaded with Au nanoparticles (0.5 wt%) revealed that Au significantly enhanced the chemical adsorption activity and the quantity of NO_2_ on the material surface. Au catalyzed the formation of more highly reactive adsorbed species at the interface, such as monodentate nitrite (characteristic IR peak at ~1108 cm^−1^) and chelating bidentate nitrite (peaks at ~1284 cm^−1^ and ~1188 cm^−1^). The energy barrier of the forming of these negatively charged adsorbed species was also lesser with the presence of Au. This alteration in surface chemistry directly resulted in a notably higher response of the Au/In_2_O_3_ sensor to low concentrations of NO (0.6–5 ppm) at 100 °C compared to pure In_2_O_3_ (Figure 6d), along with faster response/recovery kinetics. The fundamental nature of chemical sensitization, as is evident in this case, is that noble metal catalysts can increase reaction rates and selectivity, by decreasing the activation energy of important surface reaction steps (e.g., gas dissociation, oxygen activation, and intermediate formation), controlling reaction pathways, and, in the end, yielding high sensitivity and rapid-response gas sensing behavior.

### 2.3. Selectivity: Targeted Recognition and Anti-Interference Mechanisms

The enhancement of gas selectivity through noble metal modification stems from their unique chemical bonding interactions with specific gas molecules [53,54,55]. For instance, the interaction between palladium (Pd) and hydrogen (H_2_) involves dissociative adsorption, where Pd surface atoms cleave the H–H bond of H_2_ molecules [24,56]. This process arises from the distinct electronic configuration of Pd atoms, which selectively activate H_2_ without similar effects on other gases such as CO. Gold (Au), on the other hand, exhibits high selectivity toward aromatic compounds via π-complexation, where Au atoms form planar coordination structures with benzene rings. This interaction stabilizes benzene adsorption on the Au surface, while linear molecules like ethanol are repelled due to incompatible electron cloud distributions. Silver (Ag) recognizes formaldehyde (HCHO) through coordination bonds, where Ag^+^ ions form strong interactions with the carbonyl oxygen atom of HCHO. Such bonding is ineffective against molecules like acetone, which lack lone-pair donors. These specific chemical interactions significantly enhance the selectivity of metal oxide sensors toward particular gases when modified with the corresponding noble metal.

The unique selectivity enabled by Pd’s dissociative adsorption of H_2_ is clearly demonstrated in the study by Soheil Mobtakeri et al. [51]. Here, Pd modification increased the response of MoO_3_ nanowall sensors to 1000 ppm H_2_ by 754 times at 300 °C (compared to the unmodified sample), achieving a response value as high as 3.3 × 10^5^ (Figure 6d). This activation process is highly specific to H_2_, as other gases like CO cannot be effectively dissociated and adsorbed by Pd (Table 4).

On the other hand, the remarkable selectivity for aromatic compounds conferred by π-complexation is evident in the research by Tianyu Yang et al. [52]. In their work, Au-Pt bimetallic-decorated ZnO sensors exhibited a high response of 39 toward 50 ppm benzene, far exceeding their responses to other VOCs (e.g., only 8.2 for 50 ppm methanol) (Figure 6e). This selectivity originates from the specific interaction between the delocalized π-electron cloud of benzene and the d-orbitals of Au atoms, while linear or non-conjugated molecules (e.g., ethanol) cannot form equally stable complexes.

The gas sensing characteristics of SMOs functionalized with different noble metals, such as Au, Pt, and Pd, are detailed in Table 2, Table 3 and Table 4, which compiles critical parameters including target gas, operating temperature, response magnitude, and detection limit.

**Table 2:** GasNAsensitive response of PtNAdecorated SMONAbased gas sensors.

Materials/The Loading Amount/Types of Precious Metals	Target Gas	Critical Parameters (O.T. Conc. Response Tres/Tre LOD)	Refs.
ZnO/2 wt% 1:1/AuNAPt	benzene	300 °C 50 ppm 39 8/30 NA	[52]
rGONAFe_2_(MoO_4_)_3_/5 wt%/Pt	xylene	175 °C 100 ppm 41.3 NA 500 ppb	[57]
ZnO/NA/Ag	NO_2_	250 °C 1 ppm 434.3% NA 1 ppb	[58]
SnO_2_/NA/Pt	H_2_	79 °C 1000 ppm 197 < 1/20 s NA	[59]
ZnO/NA/Pt	ethanol	150 °C 50 ppm 843 4/22 min NA	[60]
WO_3_/1 at%/AuNAPt	MeSa	450 °C 50 ppm 5.4 NA 41 ppb	[61]
GONAZnO/NA/Pt	acetone	220 °C 50 ppm 150.2 4/7 s NA	[62]
SnO_2_/NA/Ga	NH_3_	275 °C 1000 ppm 5980, 115/30 s 0.1 ppm	[63]
WO_3−x_/NA/Pt	acetone	350 °C 5 ppm 43.4 NA 0.1 ppm	[64]
ZnO/NA/Pt	TEA	200 °C 100 ppm 4170 34/76 s 100 ppb	[46]
ZnO/1 at%/Pt	H_2_	150 °C 100 ppm 63.8% NA NA	[47]

**Table 3:** GasNAsensitive response of AuNAdecorated SMONAbased gas sensors.

Materials/The Loading Amount/Types of Precious Metals	Target Gas	Critical Parameters (O.T. Conc. Response Tres/Tre LOD)	Refs.
In_2_O_3_/NA/Au	n-butanol	325 °C 100 ppm 1054 8/15 s 50 ppb	[49]
SnO_2_/0.1 at%/Au	NO	130 °C 100 ppb 183 163/146 s 1 ppb	[65]
WO_3_/1 wt%/Au	NO_2_	30 °C 1000 ppb 64.19 55/24 s NA	[66]
In_2_O_3_/0.2 wt%/Au	CO	240 °C 50 ppm 18.2 37/86 s NA	[67]
WO_3_/5 at%/Au	H_2_S	400 °C 1 ppb 2.01 NA 1 ppb	[68]
SnO_2_/NA/Au	H_2_S	24 ± 1 °C 500 ppb~270% NA/126 s 2 ppb	[69]
W_18_O_49_/NA/Pd@Au	H_2_S	100 °C 50 ppm 55.5 NA NA	[70]
SnO_2_/NA/Au	DMMP	200 °C 680 ppb 1.88 25/72 s 34 ppb	[71]
Co_3_O_4_/NA/Au	acetone	250 °C 10 ppm 27.05 NA NA	[72]
ZnO/0.5 wt%/Au	isoprene	350 °C 50 ppb 42 NA NA	[73]
ZnO/NA/Au	ethanol	200 °C 50 ppm 159 NA NA	[74]
ZnO/4 wt%/Au	formaldehyde	70 °C 100 ppm 68.8 216/106 s 0.25 ppm	[75]
ZnO/exfoliated WSe_2_/0.5 wt%/Au	Benzene	25 °C 30 ppm 255.64% 40/58 s 0.1 ppm	[76]
ZnO/2 wt% 1:1/AuNAPt	Benzene	300 °C 50 ppm 39 8/30 NA	[52]

**Table 4:** GasNAsensitive response of PdNAdecorated SMONAbased gas sensors.

Materials/The Loading Amount/Types of Precious Metals	Target Gas	Critical Parameters (O.T. Conc. Response Tres/Tre LOD)	Refs.
SnO_2_/Pd/In_2_O_3_	CH_3_COCH_3_	400 °C 200 ppb 2.9 4/47 NA	[19]
ZnO/Pd@ZIFNA8	CH_4_	210 °C 500 ppm 57.9% NA NA	[77]
SnO_2_NArGO/5 at%/Pd	H_2_	360 °C 200 ppm 32.38 NA NA	[78]
nNAZnO/PdNA30 wt%Ag	CH_4_	50–100 °C 100–10,000 ppm 45–80% <1–67 s 80–270 ppm	[48]
CuCrO_2_/NA/Pd	H_2_S	150 °C 50 ppm 72.3% 35.9/182.7 s 500 ppb	[79]
ZnO/NA/Pd	H_2_	RT 50 ppm 70% 137/165 s 10 ppb	[80]
MoO_3_/NA/Pd	H_2_	250 °C 1000 ppm 3.3 × 10^5^ 379/304 s NA	[51]
NiONAZnO/0.31 wt%/Pd	H_2_	150 °C 50 ppm 65% 48/216 s 0.1 ppm	[81]
InNAZnNAO/NA/Pd	H_2_	250 °C 1% 15,900 20/51 s 100	[82]
Ga_2_O_3_/NA/Pd	NO_2_	RT 100 ppm 146.56% 12/23 s 51 ppb	[83]
ZnO/0.3 wt%/Pd@Pt	NO_2_	80 °C 50 ppb 60.3 160/230 300 ppt	[84]
WO_3_/NA/Pd	H_2_	110 °C 10 ppm 40.63 49/73 s NA	[85]
MILNA125NATiO_2_/NA/Pd	CH_2_O	RT 100 ppm 15 37/12 NA	[86]
αNAFe_2_O_3_/0.59 wt%/Pd	H_2_	300 °C 200 ppm 41,000 49/533 s 50 ppb	[87]
WO_3_ NSs/2 wt%/Pd	MustardGas	260 °C 700 ppb 8.5 9/92 s 15 ppb	[88]
ZnO/NA/Pd	CH_3_NH_2_	250 °C 400 ppm 99.5%–25 ppm	[89]

## 3. Factors Influencing Noble Metal Modification

### 3.1. Optimization of Noble Metal Loading Amount

The loading amount of noble metal is a critical factor affecting the gas sensing performance of materials. In optimizing the loading, a key threshold exists: loading that is too low leads to an insufficient number of active sites, failing to effectively catalyze reactions, whereas excessive loading readily induces agglomeration of noble metal particles, significantly reducing the effective specific surface area and potentially forming a continuous conductive metallic network [58,67]. This not only diminishes catalytic efficiency but also weakens the resistance modulation capability of the semiconductor substrate in response to gases, and may even reduce selectivity [85,90]. Therefore, the ideal loading amount must achieve highly dispersed noble metals to maximize exposed active sites while avoiding masking the intrinsic gas sensing properties of the base semiconductor material.

Our group employed high-throughput experimental technology to rapidly screen the NO_2_ sensing performance of various noble metal/In_2_O_3_ sensors (Table 5) [90]. It identified 0.5 mol% as the optimal Ag loading. This work confirms that the ideal loading is a result of the synergy between the noble metal properties, support interface, and target gas molecules, which must be determined through systematic performance testing rather than predicted by any single theoretical parameter.

**Figure 7:**
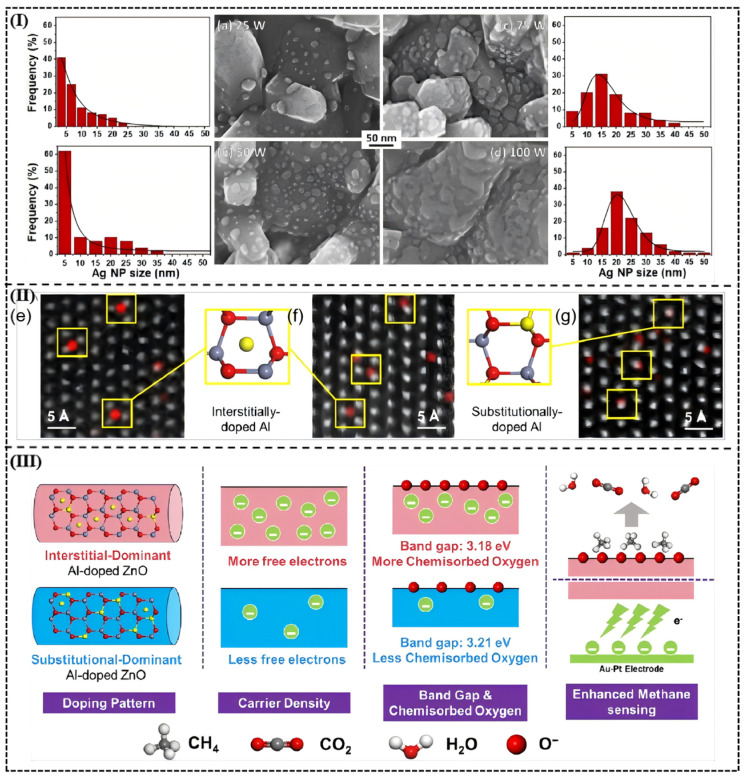
(**I**) SEM images of ZnO nanostructures with AgNPs deposited at (**a**) 25 W, (**b**) 50 W, (**c**) 75 W and (**d**) 100 W, along with their Ag NP size distribution statistics measured based on SEM images. Reproduced with permission from Ref. [58] © 2025 Elsevier B.V. All rights are reserved, including those for text and data mining, AI training, and similar technologies. (**II**) ACTEM images and Al EDS mappings of (**e**) AZO-0.1%, (**f**) AZO-0.2%, and (**g**) AZO-1%. The gray, red, and yellow balls represent Zn, O, and Al atoms, respectively. (**III**) Schematic of the mechanism underlying the enhanced CH_4_ sensing performance of interstitially Al-doped ZnO. Reproduced with permission from Ref. [91].Copyright © 2025, American Chemical Society.

A research paper by Zong-Ying Shen et al. [58] regarding Ag-modified ZnO nanorods to detect NO_2_ demonstrates how crucial the loading of the metal material is in the optimization of the sensor parameter. When sputtered using a power of 25 W which corresponds to a 0.11 at% loading of Ag, the nanoparticles had a mean diameter of 3.2 pm and a homogeneous spread (Figure 7a), thus giving high density of catalytic active sites with a sensor response of 434.3 percent at 1 ppm of NO_2_. Conversely, as the loading was increased to 0.39 at% (sputtering power 100 W) the agglomeration was substantially induced, the particle size increased to 22.4 nm (Figure 7d), and a continuous conductive network developed that hindered the diffusion of gases, which also lowered the response to 315%. This loading-dependent threshold effect shows the dynamic compensation between catalytic efficiency and dispersion; optimum loading reduces agglomeration of metals and maintains the inherent gas sensing properties of the semiconductor substrate, and makes the most efficient use of the active sites.

Noble metal loading optimization is a standard technique applied to improve the work of gas sensors. As illustrated by Chen et al. [91] in the research of our group, the effects of the careful formation of the doping locations, and, therefore, on the electronic structure can be achieved through the purposeful control of the dopant loading levels. Through a careful manipulation of NaAlO_2_ additive, the Al concentration was adjusted to the point where interstitial-dominant to substitutional-dominant doping regime was changed. Direct observation of the atomic-level doping locations was used to make the use of X-ray diffraction (XRD) and aberration-corrected transmission electron microscopy (ACTEM) with energy dispersive spectroscopy (EDS) mapping. The interstitial-doped AZO-0.2(100)-0.2 sample has also shown a lattice expansion (i.e., the (100) peak was moved to 34.34°), and substitutional-doped AZO-1(100)-0.2 sample decreased the lattice, proving that the doping mode could be controlled. ACTEM images showed that the electrons of the interstitial arrangement fit the Zn atoms with the Al atoms at the center of three faces whereas in the substitutional arrangement, an Al atom took the place of Zn atoms so that the electrons are more optimized in distribution (Figure 7II).

It was also found that using the interstitial-dominant AZO-0.2% sensor, which responded at 42.4% to 500 ppm of CH_4_, at 280 °C, was much higher than the responses of pure ZnO (20.2% response) and the substitutional-dominant AZO-1% sensor (39.2% response). In addition, the interstitial-doped sensor achieved a response time and recovery time of 3.8 and 5.0 s, respectively, which is the best, compared to the majority of doped metal oxide semiconductors.

Mechanism analysis found that interstitial doling added more free electrons (each Al atom adding three net electrons), thus making its carrier density be 2.46 × 10^20^ cm^−3^, and substitutional doling added only one net electron which amounts to carrier density being 8.46 × 10^19^ cm^−3^ (Figure 7III). Calculations using density functional theory (DFT) showed that interstitial doping reduced the band gap to 2.998 eV (as compared to 3.299 eV in the undoped ZnO) and allowed charge transfer between the conduction and the valence band. Further experiments involving oxygen temperature-programmed desorption (O2-TPD) experiments showed that the interstitial-doped sample was sixfold that of chemically adsorbed pure ZnO and therefore O_2_ oxidation reaction of CH_4_ was accelerated (CH_4_ + 4O^−^_ads_ → CO_2_ + 2H_2_O + 4e^−^) and the response time was reduced. Electronic-state modulation effect was visually represented by band structure and density-of-states diagrams.

### 3.2. Construction of Catalytic Interfaces

Noble metal and metal oxide interface engineering is needed to improve the room temperature gas sensing performance [77,84,92]. Through accurate regulation of size, morphology, and distribution of noble metal nanoparticles on the oxide surface, atomic-level manipulation of gas–molecule adsorption–reaction pathways can be attained; this will increase the electron transfer and catalytic reaction kinetics and therefore, simultaneously increase the sensor sensitivity and selectivity. This plan gives a different orientation of a traditional material design and forms basis of further interface engineering.

Based on this, interface engineering avoids classical performance bottlenecks by the synergism design of core and shell structures as well as heterojunctions [19,57,69,70,74]. The core–shell structure utilizes atomic-scale coating technology to inhibit high-temperature Ostwald ripening—a product-loss process whereby atoms on the surface of small particles are dissolved by high curvature energy barriers, diffuse through the substrate, and adsorb to large particles, thus being lost [93,94]. A thick shell is appropriate to prevent the lineages of atomic migration, protecting the dispersion and activity of the metal particles. It can also screen physically in terms of size and chemically in terms of adsorption competition based on its shell structure.

**Figure 8:**
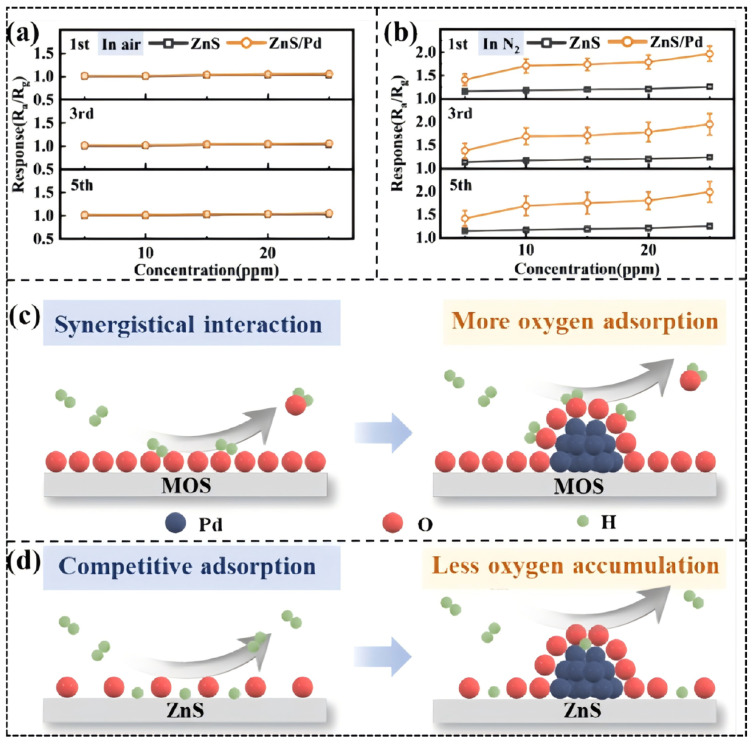
(**a**) Response of ZnS and ZnS/Pd NPs exposed to H_2_ in air; (**b**) response of ZnS and ZnS/Pd NPs in N_2_; and schematic diagram of (**c**) MOS and (**d**) ZnS-sensing process. Reproduced with permission from Ref. [92]. Copyright © 2024, American Chemical Society.

A good example of this is the Pt-shell-modified Pd-core structures of Li, Y. R. et al. [92]. Pt-shell-modified Pd-core structures are based on the high oxygen adsorption affinity of Pt (the energy of the Pt-O bond is much greater than the energy of the Pd-O bond). When exposed to air, oxygen molecules are captured and immobilized mostly by Pt at the surface, forming a localized oxygen isolation layer. This physically impedes diffusion of oxygen to the Pd core and what is more important is that this process is a screening process on a molecular level due to chemical adsorption energy differences. Small dementia (reducing, 0.289 nm in diameter) hydrogen molecules, therefore, can enter the shell to react with Pd core to give Pd-H bonds, but larger oxidizing poisoning molecules (e.g., H_2_O 0.26 nm not shown, but permeable) or organic contaminants will be excluded (Figure 8c,d). It is a dual-level filtration system, comprising physical size screening and chemical adsorption competition that allows Pd active sites to specialize in target gas reactions, eventually providing ultra-high sensitivity detection and anti-interference of hydrogen in the air compared to the situation with single-size screening.

**Figure 9:**
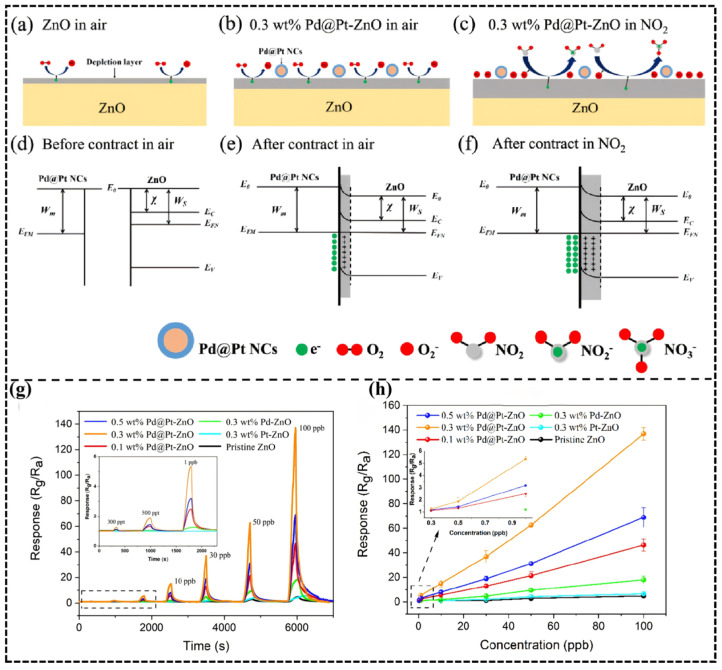
(**a**–**f**) Schematic diagram of sensing mechanism (W_M_, the work function of metal; W_S_, the work function of semiconductor; χ, electron affinity; E_0_, vacuum level; E_C_, conduction band; EFM, EFN, Fermi level; and EV, valence band); (**g**,**h**) dynamic response and response relationship to NO_2_ from 0.3 to 100 ppb of the sensors at 80 °C in 25% RH. Reproduced with permission from Ref. [84] Copyright © 2024, American Chemical Society.

Furthermore, the interface band modulation mechanism of heterojunctions enhances gas response through directional carrier migration [95]. A typical example is demonstrated in the Pd@Pt core–shell modified ZnO system studied by Chengming Sui et al. [84]: the Pt shell (work function 5.65 eV) forms a Schottky barrier upon contact with ZnO (4.5 eV), driving electron transfer from ZnO to Pt and significantly increasing the surface-adsorbed oxygen concentration. When exposed to NO_2_, its strong electron-acceptor character increases the barrier height from 0.7 eV to 1.2 eV (Figure 9a–f), causing a drastic change in resistance and achieving a high response of 60.3 (Rg/Ra) to 50 ppb NO_2_ with a detection limit at the 300 ppt level (Figure 9g,h).

Single-atom doping (e.g., with Au, Pt) has emerged as a revolutionary strategy in recent years, constructing highly efficient catalytic interfaces through atomically dispersed noble metal sites [96,97,98,99]. This approach addresses the issue of active site blocking common in traditional core–shell or heterojunction structures. By leveraging strong coordination between metal atoms and the semiconductor substrate, it forms uniform and high-density active centers, greatly improving atomic utilization and interfacial charge transfer efficiency.

**Figure 10:**
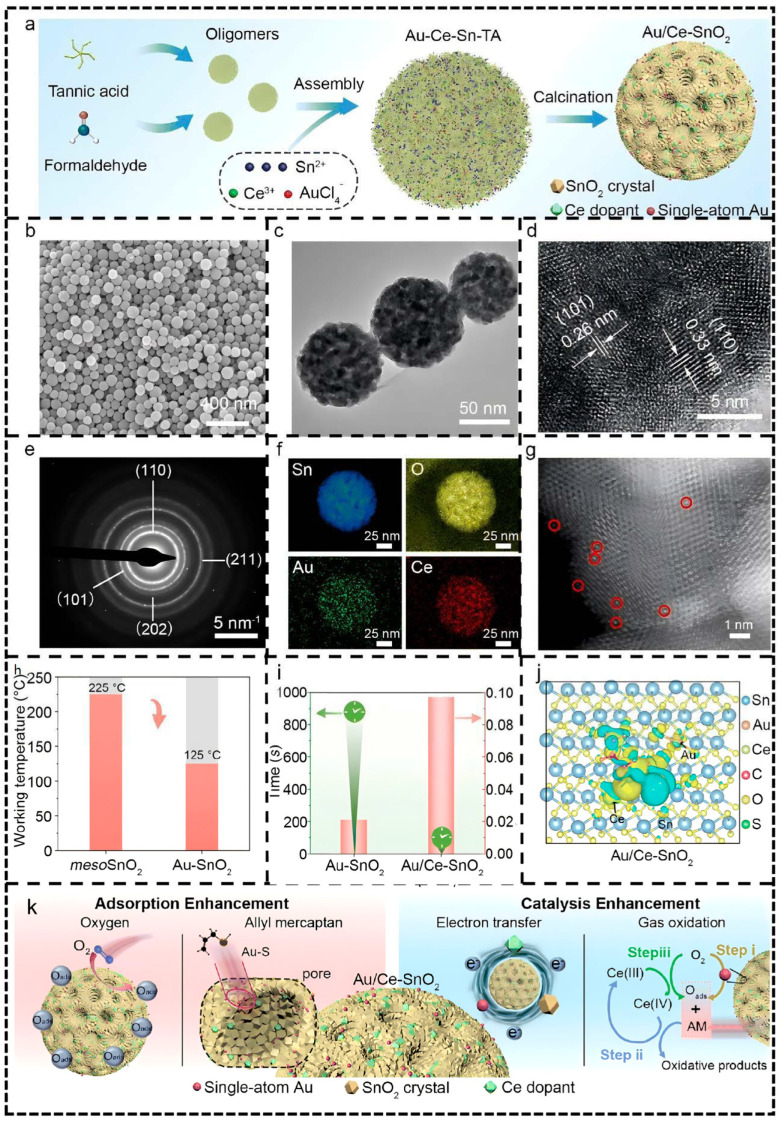
(**a**) The synthesis strategy of Au/Ce–SnO_2_. (**b**) SEM, (**c**) TEM, (**d**) HRTEM, (**e**) SAED pattern, (**f**) element mapping, and (**g**) HADDF image of the typical sample (Au/Ce–SnO_2_). (**h**) The sensitization of single-atom Au on mesoporous SnO_2_ nanospheres. (**i**) The response time and sensitivity for Au/Ce–SnO_2_ and Au–SnO_2_ materials. (**j**) The charge transfer between Au/Ce–SnO_2_ (101) and allyl mercaptan. The blue region: the consumption of charge; the yellow region: the accumulation of charge. (**k**) The proposed synergistic sensitization effects of the sensor towards allyl mercaptan (AM). O_ads_: the surface-adsorbed oxygen. Reproduced from Ref. [100] with permission from the Royal Society of Chemistry.

A study by Wei et al. [100] on single-atom Au-modified mesoporous SnO_2_ serves as a representative case: Au/Ce-SnO_2_ material synthesized via a self-templating strategy was clearly shown by HAADF imaging to have atomically uniform dispersion of Au (no agglomeration) (Figure 10g, the red circles is single-atom Au). This precise interface design enabled each Au atom to form an Au-O_2_ coordination configuration with two oxygen atoms, optimizing the adsorption and dissociation pathways of oxygen molecules.

Synchrotron EXAFS analysis further confirmed the atomic state characteristics of Au (Au–O bond length 2.02 Å), and Bader charge calculations revealed enhanced charge transfer (0.53 eV), providing direct evidence for interfacial electronic reconstruction. This atomic-level interface engineering significantly promoted the adsorption and activation of target gases (e.g., allyl mercaptan). Combined with the redox synergy of Ce doping (Ce(III)/Ce(IV) cycle), the sensor achieved ultra-high sensitivity (0.097 ppb^−1^) and an ultra-low detection limit (0.74 ppb) at 125 °C (Figure 10h). The performance enhancement mechanism can be summarized as a synergistic “adsorption enhancement–catalytic enhancement” pathway—single-atom Au preferentially adsorbs and dissociates oxygen molecules, while Ce doping accelerates target gas oxidation and electron release (Figure 10k).

Compared to traditional noble metal nanoparticle modification, single-atom interfaces avoid high-temperature Ostwald ripening, providing a theoretical and technical foundation for the next generation of low-power, high-selectivity gas sensors.

### 3.3. Illumination

In the context of gas sensing studies, the light of the sensing element is an external source of energy that alters the functioning of noble metal-modified materials in various physicochemical processes. When irradiated, the semiconductor substrate absorbs photon energy, which is initially used to excite electrons located in the valence band to the conduction band, and increases the intrinsic carrier concentration as a result. Meanwhile, light is known to promote the photocatalytic generation of surface-adsorbed oxygen species, which enhances the rate of the redox reaction between gas molecules and active sites [101,102]. It has to be said that not every type of photosensitive material can demonstrate such a great improvement in performance; its effectiveness is influenced by the ratio between the material band gap and the wavelength of incident light and ability of interfacial defect states to eliminate the recombination of electrons and holes.

Focusing on this fact, the addition of noble metal nanoparticles thereof (e.g., Au, Ag, Pd) provides a new light responsivity mechanism—surface plasmon resonance (SPR) [103,104,105,106,107]. SPR is a natural reaction of metal nanostructures of noble elements (e.g., Au, Ag, Pt) to certain optical excitation and produces an impressive localized electromagnetic field growth at the metal–dielectric interface due to the synchronization of all electrons in the specimen. This effect significantly enhances the light-harvesting efficiency of semiconductor metal oxides by synergistic interactions: metal nanoparticles are considered subwavelength optical antennas, increasing the pathlength of incident photons within the semiconductor through Mie scattering; the field produced by SPR directly stimulated the growth of the number of carriers in the semiconductor by overcoming the metal–semiconductor interface, directly enhancing the number of photoreduced carriers by non-equilibrium hot electrons produced by SPR decay. This boosts the factors leading to enhanced efficiency of carriers through the utilization of optical energy within the visible to near-infrared wavelength range, which allows photoactivated gas sensors to achieve higher concentrations of carriers under low-energy illumination to facilitate what reduces the operation temperature penalty and enhances sensitivity to trace gases. The root cause of this performance gain is the optimization of the processes of light–matter interaction as opposed to changes in the inherent band structure of the material.

A typical example can be found in the Pd-modified ZnO/rGO ternary composite material developed by our research group [108]. This study constructed 2–6 nm Pd nanoparticles on a ZnO/rGO substrate via photodeposition, forming a visible light-responsive ternary heterostructure. UV-vis spectra revealed a significant absorption peak at 470 nm, confirming the SPR effect of Pd nanoparticles, and the rGO/ZnO/Pd hybrids exhibited markedly higher absorption intensity in the visible region compared to other control samples.

**Figure 11:**
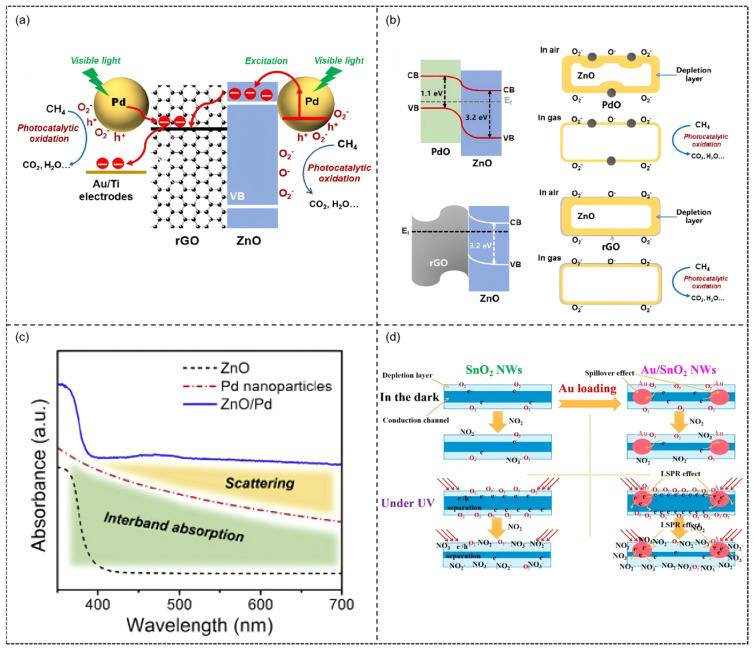
Schematic illustration of the mechanism for (**a**) photocatalytic oxidation of CH_4_, the improved electron transfer and (**b**) the multiple heterojunctions formed in the ternary rGO/ZnO/Pd hybrid under visible light illumination. Reproduced from Ref. [108] © 2019 Elsevier B.V. All rights reserved. (**c**) UV–vis absorption spectra of the Pd nanoparticles in a colloidal sus pension (red dashed line), ZnO/Pd hybrid (blue solid line) and pure ZnO reference (black dashed line). Reproduced from Ref. [109] © 2019 Elsevier Ltd. and Techna Group S.r.l. All rights reserved. (**d**) Schematic illustration of the sensing mechanism in this study. Reproduced with permission from Ref. [110]. Copyright 2022, open access.

Photoelectrochemical tests further showed that the photocurrent density of the ternary heterostructure reached 66.5 μA (at 2.0 V) under 470 nm illumination, six times higher than that of pure ZnO, indicating significantly improved charge separation efficiency. Under 470 nm visible light illumination, the sensor exhibited a high room temperature response of 63.4% to 1% CH_4_, with response/recovery times shortened to 74 s/78 s, significantly outperforming its performance in dark conditions (response only 4.1%). This enhancement is attributed to the multiple effects induced by SPR: Pd nanoparticles act as photosensitizers promoting hot electron injection into the ZnO conduction band, increasing the generation of active oxygen species (·O_2_^−^ and O^−^); rGO nanosheets serve as efficient charge transport networks accelerating electron migration; the multiple Schottky barriers formed at the ternary heterointerfaces optimize carrier separation efficiency (Figure 11a,b). Moreover, in situ FT-IR analysis demonstrated the reaction pathway of CH_4_ oxidation to CO_2_ and H_2_O, with the apparent rate constant (k) showing a positive correlation with the sensing response value (R^2^ = 0.98), establishing a quantitative relationship between photocatalytic activity and sensing performance. This work provides direct experimental evidence for the mechanism of noble metal SPR effects in gas sensing, promoting the application of room temperature-photoactivated sensors in coal mine safety monitoring.

This exemplary case demonstrates powerful synergy between electronic sensitization, chemical sensitization and plasmonic effects. The SPR of Pd nanoparticles acts as an “energy switch” that simultaneously enhances chemical catalysis and modulates charge transfer. The rational ternary design provides optimal pathways for both surface reactions and electron transport. Most importantly, the quantitative correlation between photocatalytic activity and sensing response establishes a direct link between microscopic mechanisms and macroscopic performance. This work provides a paradigm for multi-mechanism integration in high-performance gas sensors, demonstrating that deliberate synergy between different enhancement strategies yields superior results compared to any single mechanism alone.

In gas sensing research, noble metal modification and photoactivation strategies also significantly enhance performance through other mechanisms. First, the study by J. Wang et al. exemplifies the non-plasmonic photoactivation mechanism [109]. The Pd/ZnO system exhibits a characteristic absorption peak at 475 nm, corresponding to the interband transition of Pd; under 475 nm visible light irradiation (Figure 11c), Pd nanoparticles generate non-plasmonic hot electrons via this transition and inject them into the ZnO conduction band, thereby greatly increasing the room temperature response to 100 ppb NO_2_ to 160%. In contrast, the study by B. Zhang et al. highlights the role of the LSPR effect [110]. Specifically, under 365 nm UV excitation, the LSPR of Au nanoparticles induces collective oscillations of surface-free electrons, generating abundant hot electrons that are injected into the SnO_2_ conduction band, which in turn significantly raises the carrier concentration (Figure 11d). This leads to a response of 65 to 5 ppm NO_2_ at room temperature, along with markedly accelerated response/recovery speeds. These two cases reveal the different underlying mechanisms by which noble metals enhance photoactivated gas sensing.

### 3.4. Humidity

The influence of humid environments on sensing performance is particularly complex: water molecules not only compete with target gases for adsorption sites but also alter the surface hydroxyl concentration and ionic conductivity of the material, often leading to baseline drift and sensitivity degradation [111,112,113]. For instance, in unmodified SnO_2_ sensors, the response to formaldehyde typically decays by more than 60% under 70% RH.

**Figure 12:**
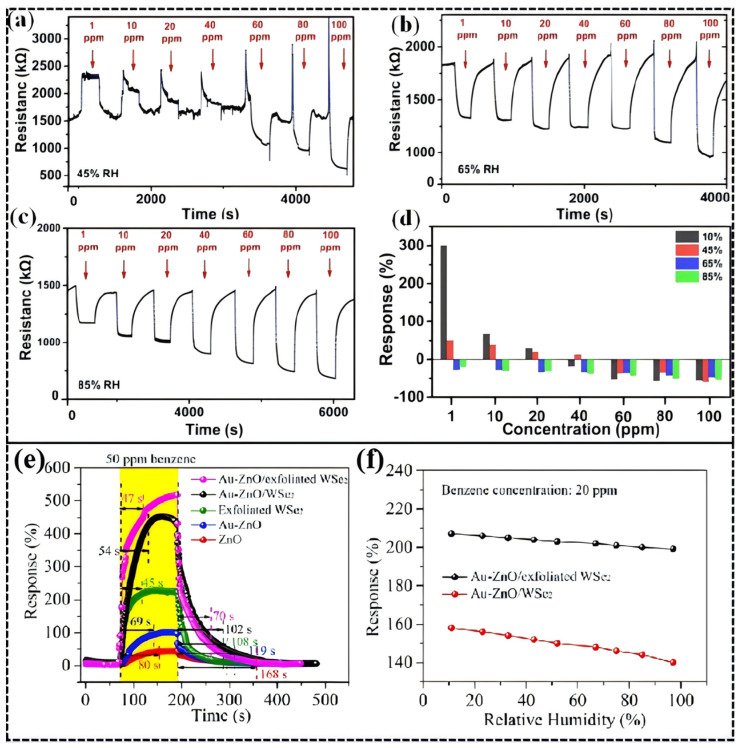
H_2_-sensing performance of the 15 s sputtered Pt/TiO_2_ in 45% RH (**a**), 65% RH (**b**), and 85% RH (**c**), and comparison of the response in different humidity conditions (**d**). Reproduced from Ref. [114] Copyright © 2021, American Chemical Society. (**e**,**f**) Sensing properties of ZnO, Au-ZnO, exfoliated WSe_2_, Au-ZnO/WSe_2_,and Au-ZnO/exfoliated WSe2 sensors toward 30 and 50 ppm benzene gas. Reproduced from Ref. [76] Copyright © 2021, American Chemical Society.

Noble metal modification offers a promising pathway to address this challenge. A study by Zhou et al. [114]. demonstrated that a Pt/TiO_2_ sensor exhibits p-type response (resistance increase) at low humidity (<30% RH) due to preferential adsorption of H_2_ on Pt forming PtH_x_, while at high humidity (>65% RH), H_2_O occupies the Pt active sites, forcing H_2_ to react directly with TiO_2_ and release electrons, resulting in an n-type response (resistance decrease). This humidity-triggered p-n transition mechanism (Figure 12a) causes a signal inversion at a H_2_ concentration threshold of 40 ppm. Although the response time increased from 25 s under dry conditions to 72 s, the sensor’s adaptability to humid environments was significantly enhanced.

**Figure 13:**
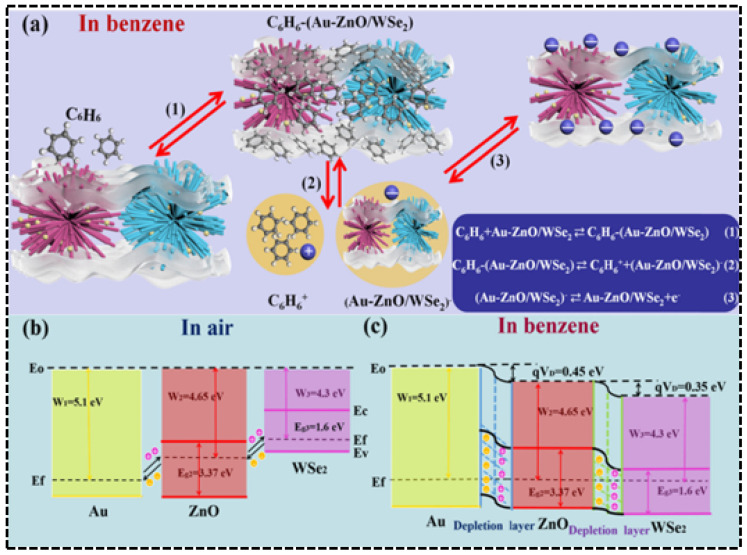
(**a**) Schematic of the sensing mechanism of Au-ZnO/exfoliated WSe_2_ composites in a benzene atmosphere. Energy band schematic (**b**) in air and (**c**) under benzene conditions. Reproduced from Ref. [76] Copyright © 2021, American Chemical Society.

The suppression of humidity interference by noble metal composite structures is also notable in benzene sensing. Research by Zhang et al. [76]. showed that a Au nanoparticle-modified ZnO/WSe_2_ heterojunction, leveraging the catalytic activity of Au and interfacial charge modulation (Figure 13a–c), achieved a high response of 520% to 50 ppm benzene at 25 °C without light illumination (Figure 12e). More importantly, as shown in (Figure 12f), when the environmental humidity increased from 11% RH to 97% RH, the sensor’s response decay was only about 10%, far lower than the >50% decay observed in traditional materials. DFT calculations further revealed that the introduction of Au significantly enhanced the adsorption energy of benzene molecules from −0.92 eV (pure ZnO) to −2.58 eV, greatly reducing the impact of competitive water molecule adsorption and highlighting the anti-interference advantage of noble metal modification in extreme humidity conditions.

### 3.5. Other Factors

In the framework of gas sensor development, solid solution materials offer new opportunities in the optimization of the sensing characteristics, as they possess rich physicochemical characteristics and internal structures, which can be tuned in a natural fashion.

Our team of researchers reported In/Fe oxide nanowires solid solutions that were fabricated through a co-spinning system utilized in a part of the composition α-(Fe_1−x_In_x_)_2_O_3_: In/Fe oxide nano-structured materials are crystal-phase oxide nanowires with this type [115]. Analysis of X-ray diffraction showed that a single material of the solid solution phase was formed when the level of In^3+^ was 10–20%; the diffractions peaks became situated at lower values of 2θ with In content and the lattice constant increasing with the substitution of the larger In^3+^ (0.092 nm) with the smaller Fe^3+^ (0.067 nm).

**Figure 14:**
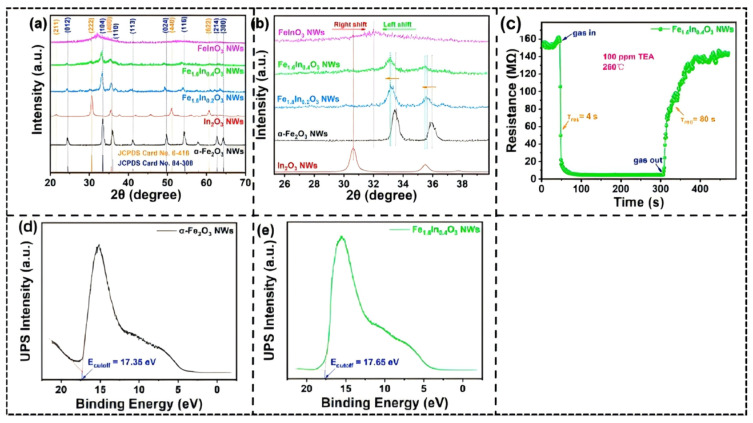
(**a**) XRD patterns of α-Fe_2_O_3_, In_2_O_3_, and three In/Fe composite NWs. (**b**) Locally amplified XRD patterns of all as-synthesized samples. (**c**) A comparison of single-cycle response–recovery transient curves of sensors based on Fe_1.6_In_0.4_O_3._ (**d**,**e**) UPS spectra (He I) of α-Fe_2_O_3_ and Fe_1.6_In_0.4_O_3_ NWs. Reproduced from Ref. [115] Copyright © 2022, American Chemical Society.

Microstructural analysis showed that the incorporation of In^3+^ had significant morphological changes on the material: Fe_1.8_In_0.2_O_3_ nanowires showed interconnected pore channels which allowed better gas permeability, compared to the amorphous regions of FeInO_3_ nanowires which hindered the movement of gases. The performance testing showed that Fe_1.6_In_0.4_O_3_ created a response at a concentration of 100 ppm triethylamine at 260 °C with the response time of 4s only (Figure 14c), an improvement from the response of pure α-Fe_2_O_3_. This improvement was achieved by means of the In doping band structure modulation, which was observed through the Fermi level value of Fe_1.6_In_0.4_O_3_ increasing by 0.3 eV above that of α-Fe_2_O_3_, thus facilitating the creation of the surface-adsorbed oxygen species.

It was also noted that formation of solid solution had reached a threshold at 20% In^3+^ content; after that, the phase of segregation was observed and regions of amorphous have formed thus deteriorating recovery properties (FeInO_3_ took 395 s to recover 77%). The study clarifies the inherent relationship between composition, structure and performance in solid solutions and can be useful in design of high-performance gas sensing materials.

**Figure 15:**
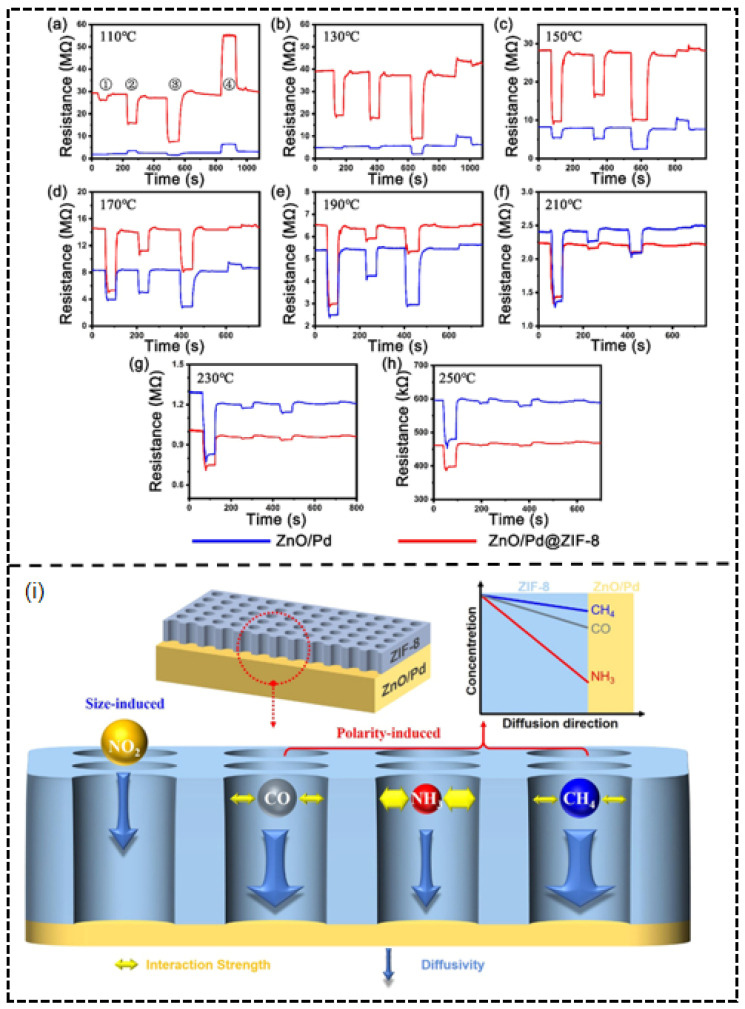
Dynamic resistance curve of the sensors based on ZnO/Pd@ZIF-8 and ZnO/Pd to ➀ CH_4_, ➁ CO, ➂ NH_3_, ➃ NO_2_ at (**a**) 110 °C, (**b**) 130 °C, (**c**) 150 °C, (**d**) 170 °C, (**e**) 190 °C, (**f**) 210 °C, (**g**) 230 °C, (**h**) 250 °C. (**i**) Schematic illustration of size and polarity-induced dual filtering effect of ZIF-8. Reproduced from Ref. [77] © 2023 Elsevier B.V. All rights reserved.

On the other hand, the study on ZnO/Pd@ZIF-8 core–shell structures reflects innovative applications of noble metal modification in traditional metal oxide systems. The ZnO/Pd@ZIF-8 core–shell structure developed by our research team exemplifies this strategy [77]. Successfully synthesized via a self-templating method, the ZIF-8 shell acts not only as a physical barrier but also achieves intelligent molecular sieving of gas molecules through its unique pore structure and chemical properties (Figure 15i). The sensor’s exceptional performance originates from the dual filtering mechanism of the ZIF-8 shell. Firstly, there is the size sieving effect: the effective aperture of ZIF-8 (~4.0–4.2 Å) physically hinders the diffusion of larger molecules like NO_2_ (kinetic diameter 4.5 Å) toward the internal ZnO/Pd sensing layer. More importantly, there is also the polarity sieving effect: for polar molecules CO (3.76 Å) and NH_3_ (2.6 Å) with kinetic diameters smaller than the aperture, their molecular polarity causes strong interaction with the ZIF-8 framework, thereby increasing diffusion resistance. In contrast, non-polar CH_4_ molecules (3.8 Å) are virtually unaffected and diffuse the fastest. Performance tests revealed that at the optimal operating temperature of 210 °C, the sensor exhibited a high response of 57.9% to 500 ppm CH_4_, with selectivity coefficients of 20.6, 11.8, and 113.1 against the interfering gases CO (50 ppm), NH_3_ (50 ppm), and NO_2_ (5 ppm), respectively (Figure 15a–h). This synergistic sieving mechanism was confirmed through in situ FT-IR and TPD experiments, which clarified the order of interaction strength between gas molecules and ZIF-8 as CH_4_ < CO < NH_3_.

## 4. Challenges and Future Perspectives

1. Lagged Research of Bimetallic/Multimetallic Synergistic Effects.

Current research heavily favors single-metal systems, leaving the synergistic effects in bimetallic/multimetallic alloys significantly underexplored, which hinders the rational design of high-performance materials [62,116]. Future efforts should focus on employing advanced characterization techniques such as in situ X-ray Photoelectron Spectroscopy (XPS) and diffuse reflectance infrared Fourier transform spectroscopy (DRIFTS), combined with theoretical calculations, to uncover synergistic mechanisms—including electronic effects, interfacial effects, and catalytic synergy—at the atomic/molecular level. The precise synthesis of novel nanostructures like core–shell, intermetallic compounds, and even high-entropy alloys will be crucial for systematically establishing composition–structure–property relationships, providing a new paradigm and a theoretical blueprint for designing sensors with high selectivity and stability.

2. An outstanding Long-term Stability and Humidity Resistance Bottlenecks.

Fluctuating environmental humidity causes baseline drift and sensitivity loss, posing a major obstacle for practical applications. Overcoming this challenge requires a combination of material innovation, device design, and intelligent algorithms—developing intrinsically hydrophobic materials (e.g., via surface fluorination) or incorporating Metal–Organic Frameworks (MOFs) as molecular sieve protective layers to enhance intrinsic humidity resistance, while advancing dynamic humidity compensation algorithms. Exploring hardware solutions that integrate sensors with micro-scale temperature control modules is promising, aiming to collectively improve long-term reliability and enable lifespan prediction under complex varying conditions through both physical isolation and advanced signal processing.

3. Lack of adequacy of recognition of complex gas interference.

In environments with multiple gas components, the selectivity of single sensors is limited, and signals are easily interfered with. Future work should emphasize the deep integration of heterogeneous sensor arrays and intelligent identification algorithms. By incorporating diverse sensing elements (e.g., MOS, conducting polymers, 2D materials) to obtain rich dynamic response fingerprints, and utilizing advanced algorithms like Graph Neural Networks (GNNs) and Temporal Convolutional Networks to decode high-dimensional, non-linear data, significant progress can be made. Furthermore, integrating physicochemical models—such as surface reaction kinetics and adsorption–desorption energy barriers—as constraints into machine learning frameworks will foster the development of highly interpretable, data-efficient physics-informed algorithms, ultimately achieving accurate identification and concentration inversion of specific target gases within complex backgrounds.

4. Material System Restrictions and Cost-Effectiveness Problems.

The high cost and susceptibility to poisoning of noble metals, coupled with a research focus limited mainly to traditional oxides, restrict material diversity. Solutions lie in promoting material innovation and a shift in R&D paradigms—vigorously developing Single-Atom Catalysts (SACs) to maximize noble metal utilization and potentially unlock unique activities; actively exploring the performance boundaries of non-precious alternatives (e.g., sulfuret [117], transition-metal carbides, nitrides [118], and of MXenes [119,120]); and leveraging machine learning-assisted high-throughput computation and screening to map the relationships between composition, structure, and properties. This approach facilitates the inverse design of new materials meeting specific application needs (e.g., high selectivity, poison resistance), accelerating the practical application and industrialization of high-performance, cost-effective gas sensing technologies.

## 5. Conclusions

In summary, this review systematically elucidates the performance enhancement mechanisms, key influencing factors, and future challenges of noble metal-modified metal oxide (MOS) gas sensors. The main conclusions are as follows:

1. Performance Enhancement Mechanisms:

Noble metals play a crucial role in supporting sensor sensitivity, selectivity and response speed through simultaneous synergistic effects of electronic sensitization–interface electron transfer, and depletion layer modulation due to work function differences, chemical sensitization—catalytic weakening of activation energy barriers of reactions with gases and surface plasmon resonance—amplification of local electromagnetic field and injection of hot electrons. Multimetallic modification systems which include Pd@Pt core–shell structures are even better refinements in reaction pathways through synergistic catalysis, and, as such, they are breakthrough performances.

2. Key Influencing Factors:
  - Loading amount: The loading (threshold ≈0.1–0.5 wt%) determines selectivity and active site density.
  - Catalytic interface: Core–shell structures (e.g., Pt-shell/Pd-core) achieve specific recognition through size sieving and chemical adsorption competition; heterojunction band modulation (e.g., Schottky barrier in Pd@Pt/ZnO) amplifies response signals.
  - External stimuli: Humidity-triggered p-n response transition (e.g., Pt/TiO_2_) and light-enhanced carrier generation (e.g., UV-Pd/Ga_2_O_3_) can dynamically optimize sensing behaviors.

3. Challenges and Perspectives:

New advances must be made in inadequately understood principles of multimetallic synergy, inadequacy of stability at high humidity (>90% RH and >60% decay), and cross-interference in multigas mixtures. Future engineering aims at performing in situ characterization methods in order to address interface processes, anti-humidity interface engineering, and intelligent decoupling of sensor arrays based on AI.

Finally, functionalized metal oxide nanostructures with noble metals have turned out to be one of the prospective methods in the sphere of gas sensing with great improvement in sensitivity, selectivity and response/recovery times. As the research and development continues, such sensors will transform different fields, such as environmental monitoring, the control of industries and personal safety. The prognosis of the noble metal-modified metal oxide gas sensor is bright and its common usage will help create the world of safety, health and sustainability.

## Figures and Tables

**Table 1:** Comparative analysis of the characteristic properties of six noble metal modifiers.

Noble Metal	Work Function	Catalytic Nature	Adsorption Preference	Poisoning Resistance
Au	~5.1 eV	Selective (CO, VOCs)	Unsaturated bonds	Good
Pt	~5.6 eV	Strong Oxidizer	Oxidation pathway	Excellent
Pd	~5.1 eV	Excellent Hydrogenation	Hydrogen (H_2_)	Moderate (S-sensitive)
Ag	~4.7 eV	Medium-Temp Oxidation	O-/S-species (NO_2_, H_2_S)	Poor (S-sensitive, sinters)
Rh	~4.9 eV	High-Temp Reactions	N-/O-containing molecules	Excellent
Ru	~4.7 eV	Strong Oxidizer	Oxygen activation	Good

**Table 5:** Performance comparison of noble metal-modified In_2_O_3_ sensors to 5 ppm NO_2_.

Noble Metal	Optimal Loading, mol%	Response, Rg/Ra	Optimal Operating Temperature, °C
Ag	0.5	923.6	50
Ru	0.5	710.7	50
Pd	0.5	617.3	50
Rh	0.5	617.3	50
Au	0.3	560.6	50
Ir	0.2	184.6	50
Pt	0.5	148.7	50
Pure In_2_O_3_	-	160.5	75

## Data Availability

No new data were created or analyzed in this study. Data sharing is not applicable to this article.
